# Reproductive justice in patient care: tackling systemic racism and health inequities in sexual and reproductive health and rights in Canada

**DOI:** 10.1186/s12978-022-01328-7

**Published:** 2022-02-16

**Authors:** Karine Coen-Sanchez, Dina Idriss-Wheeler, Xaand Bancroft, Ieman M. El-Mowafi, Abdiasis Yalahow, Josephine Etowa, Sanni Yaya

**Affiliations:** 1 School of Social Sciences, University of Ottawa, Ottawa, Canada; 2 Interdisciplinary School of Health Sciences, University of Ottawa, Ottawa, Canada; 3 NorImpact, Stavanger, Norway; 4 School of Nursing, University of Ottawa, Ottawa, Canada; 5 School of International Development and Global Studies, Faculty of Social Sciences, University of Ottawa, 120 University Private, Ottawa, ON K1N 6N5 Canada; 6 The George Institute for Global Health, Imperial College London, London, UK

## Background

Systemic racism in Canada’s healthcare system continues to contribute to sexual and reproductive health and rights (SRHR) inequities for Indigenous, Black, and womxn of colour. We continue our series on Reproductive Justice (RJ) in *Reproductive Health*, by highlighting the issue of systemic racism in the context of Canadian SRHR healthcare provision and patient care. In this commentary, we broadly discuss the macro policy and meso organizational structures that contribute to SRHR inequities among racialized individuals and communities. In “*Structural Racism, Institutional Agency, and Disrespect”*, as argued by Pierce [1], to fully comprehend systematic racism, we must observe the power dynamic, and this “must be understood in terms of injustice rather than disrespect”. This “involves giving a fuller account of how institutions are related to the beliefs, actions, and intentions of individuals, and how they can come to embody a certain kind of agency” [1]. This helps us understand racism from a macro perspective, which can then be followed by an analysis at the institutional and individual levels.

These structures, rooted in colonial practices and racial oppression, include Federal and Provincial/Territorial policies that set the stage for educational systems (i.e., admission processes, training, and licensing of health care professionals) and healthcare systems that perpetuate racism experienced by Indigenous, Black and people of colour (IBPOC) at point of care. By design, these macro level structures facilitate opportunities for the dominant group, thereby reinforcing white privilege throughout meso level institutions and organizations. At the individual level, we provide examples of the lived experiences of racialized groups in Canada who face racism daily within the healthcare system, leading to worse SRHR health outcomes compared to their white counterparts [2]. Once again, we call for policies and programs designed with a Reproductive Justice lens in Canada; we need to dismantle current oppressive structures and create systems of delivery that acknowledge and understand lived SRHR experiences of IBPOC communities [3].

### Acknowledging history of colonialism and slavery: how social systems and power structures lead to IBPOC health inequities

In her beautifully written seminal piece, Dr. Camara Jones [4] provides a clear theoretical framework outlining key levels of racism to explain race-associated health disparities in the United States. Dr. Jones explains institutionalized racism as the “differential access to goods, services, and opportunities of society by race…[this] includes differential access to quality education, sound housing, gainful employment, appropriate medical facilities… differential access to information (including one’s own history), resources [wealth, organizational infrastructure], voice [voting rights, representation of government, control of media]” [4]. Disparities in socioeconomic status between races in the U.S. originate from a history of injustice, perpetuated by “contemporary structural factors” [4]. In Canada, the stories and history of our Indigenous peoples, Black communities, and other racialized groups, echo those of the United States.

The historical accounts of colonization and government assimilation of Indigenous Peoples into Western Society have been documented and continue to surface. Colonization lead to Indigenous peoples’ losing their cultural practices, language, traditional medicines and ways of knowing and being [5]. With assimilation—the process by which Indigenous peoples were forced into the prevailing Eurocentric culture—health inequities grew while traditional healing practices were forcibly replaced by “patriarchal healthcare systems” [5]. The impact on health of Indigenous peoples has been atrocious, leading to conditions and diseases that had not been there prior to colonization—examples include tuberculosis, alcoholism, diabetes, mental health issues, cardiovascular diseases, cancer, violence, obesity [5]. The country has only recently acknowledged its history with Indigenous people as it grapples with the Truth and Reconciliation movement towards a renewed nation-to-nation relationship [6].

Before the Underground Railroad that helped enslaved Black people in the U.S. escape north, Canada had a long history of slavery that barely exists in history books or in Canadian classrooms. Marcel Trudel documented slavery in Canada during the French regime (1629) that continued until the abolition of slavery by the British (1834) [7]. Trudel catalogued over 4000 Aboriginal and Black slaves exploited between the seventeenth and nineteenth century [7]. Western colonization and slavery contributed to the expansion of the concept of race. Racialization propelled the ascription and prescription of a number of traits that contributed to the exploitation and domination of certain racial groups [8]. In conjunction, an increase in violence was justified and reinforced by a racist penal system [9]. Denying this history maintains the status quo. Accordingly, resulting cultural stereotypes and economic oppression continue to perpetuate the social construct of race [10]. Oppression is influenced and maintained by material relations and shaped by capitalism and economic exploitation. This dialectical relationship is at the heart of a capitalist society, where material and economic structures of a given society contribute to the rise of ideas and ideologies used to justify and legitimize racism.

Founding director of the Institute of the Study of Canadian Slavery—Dr. Charmaine Nelson—discussed the need for knowledge of our complex and difficult history, “in those places where they’ve been doing this work for decades [US], they’re studying mortality rates…experiences of fertility and maternity of enslaved females” [11]. In Canada, we must acknowledge that the structural basis of these power and socioeconomic differences between races are rooted in a history of slavery and oppression, perpetuating injustices, and leading to health inequities of racialized groups. We can then educate to inform policies, programs, and interventions that dismantle institutionalized racism.

Many disciplines, including social epidemiology, sociology, and political sociology, have generated theoretical and sociological perspectives illustrating race as a system of categorization. A system where “differential relations between people and their institutions…would raise or lower the odds of different turning points [i.e., access to healthcare, education, employment] and of different elements of the population depending on physical properties of the elements themselves [Black, Indigenous, white, gender]” [12]. Furthermore, race and racism are ‘‘inextricably linked’’ [13]. That is, racism determines the social treatments received by various racial groups. It is a means of justifying treatment exhibited by social institutions for incarceration, education, healthcare, and employment. As Bonilla-Silva [14] discussed, this division generates superiority and validates white Europeans’ dominance over racialized groups. Michael Omi and Howard Winant [15] systematically examined race as a socially constructed identity, highlighting how racial categories are determined by social, economic, and political forces. How does this translate for us in Canada at the macro level of organization and how does it affect SRHR outcomes for racialized communities?

Canada’s Federal and Provincial/Territorial institutions responsible for making decisions regarding provision of healthcare to society—(i.e., healthcare system organization, funding and associated priorities, education of healthcare professionals providing care, hiring practices) are based on the British colonial system. The lenses through which decisions are made at the policy level (i.e., institutionalized) are guided by the dominant Eurocentric perspective, thus, those who are not in the dominant group tend to be negatively impacted in terms of access to opportunities. This, in turn, generates an unequal distribution of socioeconomic success and better health outcomes among white communities compared to Indigenous, Black or people of colour. Additionally, there are large complex forces at play—capitalism, globalization, medicalization, neoliberalism—contributing to systemic oppression of racialized communities and leading to poorer overall health [16]. In Canada, healthcare is a fundamental right with legislated principles of public administration, comprehensiveness, universality, portability and accessibility [17]. However, it is not immune to systemic racism, thus, creating divergent realities of “equity” that do not uphold the principles of the Canada Health Act for many of the racialized segments of society. As noted by Zachariah [18], “universality is constructed around notions of whiteness and has to be considered in the context of how this leads to health disparities and denial of equal access...unequal access outcomes and health disparities are a social-economic and social-psychological manifestation of racism as a social determinant of health”. This is particularly true for Indigenous, Black and womxn of colour who have experienced disparities in health outcomes compared to their white counterparts [19–21].

Institutionalized racism has normalized practices, laws and customs that inherently disadvantage less dominant groups. For example, many health care professions in Canada providing patient care are self-regulated (i.e., medicine, nursing, midwifery, rehabilitation), that is, the profession governs itself, outlining necessary competency, licensing and disciplinary regulations, with the primary purpose of providing care and protecting the public from harm [22]. However, there is a lack of racialized voices in leadership positions in regulatory and professional healthcare administrative organizations, perpetuating inaction and resulting in harmful health outcomes for IBPOC communities, including those who work in the healthcare system. Unconscious and conscious biases are pervasive in healthcare and educational systems. These unintentional, or intentional, cognitive biases, when unchecked, perpetuate inequities that manifest beyond patient-clinician relationships and health, into practices such as hiring and promotion [23], thus impacting opportunities for IBPOC to be part of the self-regulating administration, and maintaining the status quo. As we will discuss later, these dynamics influence individual outcomes (i.e., health, education, employment, opportunities for advancement) through the organizations and institutions responsible for delivering point of care services (i.e., hospitals, clinics) and educating health professionals (i.e., colleges and universities). It is therefore necessary to examine past and current debates on race and racism within a scientific and juridical framework to develop social equality policies and mutually constitutive processes.

Adopting “a reproductive justice lens helps us explore our history by revealing the impacts that government strategies have on the lives of individuals and communities over time” [3]. Furthermore, it helps explain people’s individual reproductive capacity that result from intersecting factors—“class, race, gender, sexuality, status of their health, and access to health care.” [3]- and inform appropriate, culturally relevant SRHR health care to IBPOC communities. The Canadian Race Relations Foundation Panel on racism as a social determinant of health, encouraged the “creation and promotion of Canadian narratives which bring to light the Canadian story of race and health” [18]. As Elizabeth McGibbon [15] outlines in *Oppression: A social determinant of health*, politicizing health creates links between systemic power structures and health, affecting health across the lifespan. Understanding and recognizing the social determinants of health is crucial and aligns with a reproductive justice framework for SRHR. Additionally, as Ross and Solinger [3] state in *Reproductive Justice: An Introduction,* “reproductive safety and dignity depended on having the resources to get good medical care and decent housing, to have a job that paid a living wage, to live without police harassment, to live free of racism in a physically healthy environment—all of these (and other) conditions of life were fundamental conditions for reproductive dignity and safety—reproductive justice—along with legal contraception and abortion” [3].

### Systemic racism in educational and healthcare institutions

French sociologist Pierre Bourdieu discussed the concept of cultural powers and race. Bourdieu outlined how systems of law, education, and employment can constitute a racist regime designed to uphold white supremacy and subjugate people of colour [24]. Representations of a particular race or ethnicity are based on forms of knowledge that risk giving an erroneous orientation to the knowledge produced and passed down (i.e., to and by health professionals). In this section, we focus on two key systems involved in SRHR care provision—healthcare (i.e., hospitals, clinics) and institutions involved in educating, training, and regulating health professionals (i.e., universities, colleges, health professional and regulatory bodies). We discuss how IPBOC, and other marginalized populations face a myriad of barriers and racisms when entering healthcare professions, when they become successful, and throughout their careers. This contributes to a lack of diversity and inclusion among health care providers in Canada, reducing culturally competent, culturally safe, and informed care, which in turn, contributes to IBPOC health inequities, particularly in SRHR.

Race-based data in Canada is limited, therefore, we only have anecdotal, fragmented, and sparse statistics to illustrate the lack of diversity in race and gender among healthcare professionals, their educators, and regulators. For instance, based on a crude analysis of websites, leadership in the schools of medicine which train doctors across Canada is predominantly white (90%), about 1/3rd female, and a 1/10th racialized [25]. A recent Canadian study called for “making medical leadership more diverse” [26]. In nursing, a profession that is largely female, leadership at the professional and regulatory organizations continues to be predominantly white, and men tend to sit at the top of the hierarchical decision-making chain [27]. Similarly, medical professional associations and regulatory authorities lack diversity with little to no representation in leadership by Indigenous, Black or people of colour [28]. With the lack of diversity in healthcare leadership and providers, how can the system of frameworks, funded research, competencies, and awards reflect the needs or lived experiences of IBPOC healthcare students and professionals? If the system is designed by a dominant group, it reflects the dominant group’s way of framing healthcare, education within healthcare and the profession. This leads to continued systemic inequities that reduce opportunities for members of racialized communities to enter the healthcare profession and provide care to their communities. Even if they succeed to enter the health professions, epistemic racism discredits their knowledge and constraints their ability to influence the healthcare decisions especially in the context of racialized communities [29].

A faculty member’s position and subjectivism contribute to the discourse of racial identities, and when predominantly held by white faculty, promotes a discursive construction of knowledge based on colonial power. These influence and reproduce the social representation of racial stereotypes in the knowledge developed and taught at post-secondary institutions. Faculties are not racialized and the colonial lens disregards contributions to SRHR curriculum from IBPOC pioneers, whereby research and education presented to future SRHR practitioners is based on white healthcare providers and patients. Increasing the diversity of faculties will help reduce racist knowledge and incorporate lived experiences of IBPOC.

Wildman and Davis [30] argued that “to call someone racist ignores the system of domination that the person is made part of, treating it as his or her sole responsibility, as a result leaving the real culprit untouched”. We should, then, call all levels of racism out to illuminate the important interconnections across micro, meso and macro levels that solidify the all-encompassing nature of racism we see IBPOC communities, health professionals and academics face daily. It is important to emphasize how racist actions at the individual or interpersonal level are critically interwoven with structural and systemic racism—the collective-emotional aspects of ‘racial orders’ [31]. Structural racism operates through the interpersonal, not outside of it; structural racism shapes the spaces of everyday racism and is itself also an outcome of cumulative patterns of everyday racism [32]. Fundamentally, the privileged social capital of the racial category of whiteness, is based on exploitation and oppression, which generates unequal economic and social conditions for racialized communities.

Unfortunately, in Canada, IBPOC have significantly higher rates of unemployment, lower income, and lower rates of post-secondary education [33, 34] compared to their white counterparts. Therefore, IBPOC communities face several barriers to entering post-secondary systems and healthcare professions because of intersecting oppressions. Existing conditions reduce likelihood of opportunities to succeed in those arenas. For example, in Canada, medical students are more likely to come from high-income households and have highly educated parents who are in professions or high-level managerial positions than the general Canadian population [35]. In terms of ethnicity as self-identified by respondents, majority tend to be white (73%) with a much lower number of Black (2%) and Indigenous (3.5%) students [35]. To enter training in health professions requires excellent marks in secondary and/or subsequent undergraduate tenure, scholarships, awards and volunteer or other opportunities (i.e., research). The system, thus, sustains privilege where students who can afford to concentrate on their studies (i.e., parents assist in finances) as they are more likely to belong to dominant power groups and know how to maneuver within the structures they dominate. This creates advantageous social capital where family networks are leveraged, providing opportunities (i.e., volunteer/ work in a laboratory in the summer) to enter, advance and succeed within health professions (i.e., application, scholarships, awards). As we discussed in some detail in our second article, IBPOC students face racism as well as wealth, education and social capital disparities, impacting their educational experience and creating barriers to their academic and professional success [36, 37]. In the current state, and with a lack of diverse faculty and “lip services” around equity policies that are clearly failing in higher education institutions [38], academic and training systems are perpetuating white privilege in education and, subsequently, healthcare professions.

Several Canadian Universities in the early 1900s banned or placed restrictions on admissions of Black medical students based on Canadian and American medical school ratings published in the American Medical Association report, [39]. The Flexner Report (1910) suggested Black medical students are better off studying basic hygiene practices than medicine, and for close to 50 years, Black students were banned from Canadian and American medical schools. Unfortunately, the nursing profession was not immune to racial segregation where Black, mostly womxn, were not permitted to attend nursing programs in Canada and told to go to the US until the 1940s when Canadian universities were pressured by community organizations [40]. Similarly, Indigenous medical students report experiencing intentional and unintentional racial micro-aggressions [41]. Once again, historical examples of oppression of marginalized groups and womxn, perpetuating a toxic culture in health education and professions.

With an established history of racism experienced by Black medical and nursing students, the present situation has barely evolved, as many report toxic learning environments making it difficult to enter and succeed in a health profession. The curriculum continues to be developed by white scholars, as the mainstream culture, and is based on normative white research, usually conducted by and with white participants. It is not surprising that research continues to illustrate the lack of cultural competence in health care training with negative health outcomes of IBPOC communities. We recognized, more recently, Canadian universities have begun to work towards changing admissions policies and curriculums. The University of Toronto accepted 24 Black students in the faculty of medicine for the graduating class of 2024—the largest in Canadian history, and Queen’s University is now incorporating the history of the ban into their first-year medical students curriculum [42].

Unfortunately, even when IBPOC health professionals are successful and enter health professions, they continue to face racism throughout their careers. They advocate for their racialized patients who suffer the injustices of systems that deny care based on discrimination and racism. The “colour blind” claim denies inequities of structures and systems that historically favour white Canadians and “ignores impacts of colonization and residential school systems” [43].

As discussed in detail in our article addressing reproductive justice in health research [36], Canada is hesitant to collect race-based and disaggregated data, thus, we have difficulty accessing statistics demonstrating inequity in Canadian healthcare services and education. The lack of data on racialized professors, students, and health professionals in the health sciences in Canada makes it difficult to demonstrate the lack of diversity in key policy decision-making positions. For example, any policy level decisions made regarding womxn’s sexual and reproductive health approaches in a clinical space are made at a Federal and/or Provincial/Territorial levels where it is estimated less than 1/4th of Federal ministers of health have been womxn. Of those, only one was First Nations and one a visible minority. Similarly in Ontario, the most populous and diverse province in Canada, 1/5th of health ministers were womxn and none who were visible minorities [44]. This leaves racialized communities, particularly womxn, with no voice in the SRHR decision making space. A key aim of the Reproductive Justice movement is to encourage womxn and girls to be “active agents of change…creating opportunities for new leaders to emerge within communities” [45, 46] to participate and advocate for appropriate health and patient care by their IBPOC communities.

### SRHR patient care for IBPOC communities in Canada—we are failing our users and providers

To understand IBPOC sexual and reproductive health experience and outcomes, we must examine intersecting factors arising from oppressive colonial policies and institutional structures. Reproductive Justice outlines “how all people experience their reproductive capacity according to multiple intersecting factors including class, race, gender, sexuality, status of their health, and access to health care” [3]. Similarly, Black Mamas Matter Alliance highlight that we must provide care that matches the needs of IBPOC womxn by considering the “intersecting oppressions that cause trauma and impact [IBPOC reproductive] health at various levels” [47]. Canadian scholar, Elizabeth McGibbon [15], reinforces how intersectionality and existing systemic power structures link racism to social determinants of health and “create and reproduce [intergenerational] poor health” for IBPOC communities. Even with established literature on negative SRHR experiences and outcomes of IBPOC womxn, systemic racism is pervasive in healthcare institutions. Awareness is lacking, discrimination abound, and implicit biases ongoing among healthcare providers, as IBPOC womxn continue to face morbid SRHR inequities [19, 48–50].

Implicit biases “explain a potential dissociation between what a person explicitly believes and wants to do (e.g. a health professional wanting to treat everyone equally) and the hidden influence of negative implicit [unconscious, uncontrollable] associations on [his/her/their] thoughts and actions” [51]. For example, a doctor perceiving a patient belonging to a particular racial group as drug-seeking or predisposed to becoming an addict when they complain of pain [43]. Racial implicit biases among healthcare professionals continue to disenfranchise vulnerable patients. In a recent Canadian study, Mahabir et al. [1] identified experiences of everyday racism by racialized health care users [2]. They discussed five clusters outlining racial and class discrimination, feeling dehumanized as a patient, issues of negligent communication, professional misconduct and unequal access to healthcare services [2]. Black Canadian womxn have gone undiagnosed, subject to racist and negative treatment, left out of discussions about their own reproductive health and their concerns dismissed by healthcare providers [48]. In a recent investigation in the B.C. healthcare system, 84% of Indigenous people reported experiencing discrimination in the healthcare system and less likely to seek medical treatment [52], increasing their chance of negative health outcomes. The death of Joyce Echaquan is a staggering example of medical racism that lead to a preventable death [53]. Ms. Echaquan died of a pulmonary edema after being mocked and insulted by medical staff because she was Indigenous, failing to properly medically assess the Atikamekw mother of seven in September 2020 [53]. Much of the work to date on the differential response of underrepresented groups is concentrated on chronic diseases (i.e., cardiovascular disease, asthma, cystic fibrosis, diabetes) and medical treatments, with little recognition of institutionalize racism and its impact on health outcomes, and even less consideration of SRHR of womxn in Canada.

A complicated relationship exists between Black womxn and the healthcare system—stemming from a history of racism and medical experimentation on Black people—and leading to mistrust and resultant negative experiences that contribute to their poor SHRH outcomes [54, 55]. In terms of SRHR, examples include, not being listened to during pregnancy and birthing, not appropriately screened for cervical or breast cancer, as well as being mis-or undiagnosed for reproductive health conditions (i.e. fibroids) [19, 49]. Similarly, Indigenous womxn in Canada have experienced, and continue to experience, reproductive injustice and unmet needs in terms of their SRHR; examples include sterilization (i.e. coercion into long-term use of contraceptives, tubal-ligations, abortions) as well as Canada’s forced birth travel experienced by Indigenous womxn living in rural and remote areas [50, 56]. We must also consider the ‘reproductive injustice’ of federally incarcerated womxn. At present, SRHR of federally incarcerated womxn in Canada—42% of whom are indigenous, many of whom have young children and are of reproductive age [57]—can be characterized as unconstitutional. Necessary health services are either unavailable (screening for breast, cervical uterine cancers even though at higher-risk) and treatment is dismal [57]. Over-incarceration, separation from children and lack of autonomy regarding SRHR decisions, reinforces intergenerational trauma and perpetuates the existing health and social inequities faced by Indigenous communities.

We need “anti-racist policies that move beyond cultural competence policies [taught in mandatory workshops that fall on deaf ears] and towards addressing the centrality of unequal power social relations and everyday racism in the health care system” [2]. A Reproductive Justice lens allows us to look at our histories which call “attention over and over to the vulnerabilities of people without institutionalized power…[who] lost their fertility to coercive, race-based sterilization programs” among other atrocities [3]; this cannot continue to happen to our racialized womxn in Canada.

### Adopting a reproductive justice framework in patient care

Reproductive Justice presents clinical health professionals with the appropriate framework to “connect family planning and other aspects of sexual and reproductive health with the disparities and complexities” that impact IBPOC womxn in Canada [58]. The framework, initiated by womxn of colour, draws on the history of slavery, civil rights, coercion regarding sterilization and contraception [58, 59]. Now the framework is used by indigenous womxn, womxn of colour and trans people to fight for reproductive social justice that considers power systems, intersecting oppressions, and ensure most marginalized people access resources [46]. Health care professionals must recognize that health disparities suffered by Indigenous populations in Canada are “*linked to the socioeconomic marginalization of indigenous communities”* stemming from a history of colonization [60]. Allan and Smylie [54] emphasize the fundamental role “colonization, racism, social exclusion and the lack of self-determination” play in the staggering disparities between Indigenous and non-indigenous people’s health [61]. Healthcare providers must understand how colonization resulted in the current health status of Indigenous peoples across Canada [62]. Knowing and acknowledging our history allows us to reveal the impacts of government strategies on the lives of racialized individuals and communities over time [3].

The current pandemic only highlighted existing health inequities faced by IBPOC communities and their concern regarding the health system. Trust in the Canadian healthcare system must be built through informed health care providers, culturally appropriate healthcare delivery, and prioritized programs that address socioeconomic determinants affecting sexual and reproductive health and rights. Knowledge of a history rooted in slavery for Black womxn, colonialism of Indigenous womxn and continued racism for IBPOC womxn, and intersections with the social determinants of health, play a key role in understanding the inequities that lead to poor SHRH outcomes. Especially important for the success of future reproductive health studies is increasing the number of Indigenous, Black, and racialized midwives, nurses, physicians, and researchers who understand the potential health impacts of systemic racism on individual and population level health outcomes. They can champion change within and across the healthcare system to ensure equitable SHRH for IBPOC communities.

## Abbreviations

SRHR: Sexual and reproductive health and rights
IBPOC: Indigenous, Black and people of colour
U.S.: United States

## References

[1] Pierce AJ (2014). Structural racism, institutional agency, and disrespect. J Philos Res..

[2] Mahabir DF, O’Campo P, Lofters A, Shankardass K, Salmon C, Muntaner C (2021). Experiences of everyday racism in Toronto’s health care system: a concept mapping study. Int J Equity Health.

[3] Ross L, Solinger. Reproductive justice: an introduction: Ross, Loretta, Solinger, Rickie: 9780520288201: books—Amazon.ca. 1st ed. California: University of California Press; 2017. p. 360.

[4] Jones CP (2000). Levels of racism: a theoretic framework and a gardener’s tale. Am J Public Health.

[5] MacDonald C, Steenbeek A (2015). The impact of colonization and western assimilation on health and wellbeing of Canadian aboriginal people. Int J Reg Local Hist.

[6] Government of Canada; Crown-Indigenous Relations and Northern Affairs Canada. Truth and Reconciliation Commission of Canada. 2015 (cited 2021 Nov 15). https://www.rcaanc-cirnac.gc.ca/eng/1450124405592/1529106060525.

[7] Trudel M. Canada\’s forgotten slaves: two hundred tears of bondage. 1st ed. 2013 (cited 2021 Dec 3). p. 255. http://www.vehiculepress.com/q.php?EAN=9781550653274.

[8] Fanon. The Pitfalls of National Consciousness by Frantz Fanon. 1961 (cited 2021 Dec 5). https://www.marxists.org/subject/africa/fanon/pitfalls-national.htm.

[9] Robinson C. Black marxism. 2nd ed. London: The University of North Carolina Press. 2000 (cited 2021 Dec 5). p. 480. https://uncpress.org/book/9780807848296/black-marxism/.

[10] Du Bois WEB. The conservation of races: 1897. In: The Problem of the Color Line at the Turn of the Twentieth Century. Fordham University Press; 2014 (cited 2021 Dec 3). 10.5422/fordham/9780823254545.001.0001/upso-9780823254545-chapter-003.

[11] McKinley S. What we don’t know about the history of slavery in Canada—and why we don’t talk about it. The Toronto Star. 2021 (cited 2021 Nov 15); https://www.thestar.com/news/canada/2021/01/31/what-we-dont-know-about-the-history-of-slavery-in-canada-and-why-we-dont-talk-about-it.html.

[12] Beckfield. Political sociology and the people’s health—Jason Beckfield; Nancy Krieger—Oxford University Press. In: Krieger N, editor. New York: Oxford University Press; 2018 (cited 2021 Nov 9). p. 173. https://global.oup.com/academic/product/political-sociology-and-the-peoples-health-9780190492472?cc=us&lang=en&.

[13] Morris A. The Scholar Denied by Aldon Morris - Paperback - University of California Press, 1st ed. Oakland, California: University of California Press; 2017 (cited 2021 Dec 2). p. 320. https://www.ucpress.edu/book/9780520286764/the-scholar-denied.

[14] Bonilla-Silva E (1997). Rethinking racism: toward a structural interpretation. Am Sociol Rev.

[15] Omi M, Winant H (1994). Racial formation in the United States: from the 1960s to the 1990s.

[16] McGibbon E, editor. Oppression: a social determinant of health. National Collaborating Centre for Determinants of Health; 2012 (cited 2021 Nov 17). https://nccdh.ca/resources/entry/oppression-a-social-determinant-of-health.

[17] Legislative Services Branch. Consolidated federal laws of Canada, Canada Health Act. Justice Laws Website. 2017 (cited 2021 Nov 30). https://laws-lois.justice.gc.ca/eng/acts/c-6/page-1.html.

[18] Zachariah M. Racism as a determinant of Health—panel discussion. Canadian Race Relations Foundation (cited 2021 Nov 17). https://www.crrf-fcrr.ca/en/resources/research-projects/item/23616-racism-as-a-determinant-of-health.

[19] Chia K. How disparities in health care hurt Black and Indigenous peoples | The Peak. The Peak. 2020 (cited 2020 Dec 22). https://the-peak.ca/2020/09/how-disparities-in-health-care-hurt-black-and-indigenous-peoples/.

[20] Gunn BL. Ignored to death: systemic racism in the Canadian Healthcare System. EMRIP the Study on Health; 2016 (cited 2021 Nov 8). https://www.ohchr.org/Documents/Issues/IPeoples/EMRIP/Health/UniversityManitoba.pdf.

[21] Nnorom O, Findlay N, Lee-Foon NK, Jain AA, Ziegler CP, Scott FE (2019). Dying to learn: a scoping review of breast and cervical cancer studies focusing on black canadian women. J Health Care Poor Underserved.

[22] Schultze R (2007). What does it mean to be a self-governing regulated profession?. J Propert Tax Asses Admin..

[23] Marcelin JR, Siraj DS, Victor R, Kotadia S, Maldonado YA (2019). The impact of unconscious bias in healthcare: how to recognize and mitigate it. J Infect Dis..

[24] Bourdieu P (1990). The logic of practice.

[25] Association of Faculties of Medicine of Canada (AFMC). Canadian Medical Education Statistics 2019—section A: AFMC Faculties of Medicine Data. AFMC; 2019. https://afmc.ca/sites/default/files/pdf/CMES/CMES2019-SectionA_EN.pdf.

[26] Ruzycki S, Franceschet S, Brown A. Making medical leadership more diverse—ProQuest. (cited 2021 Dec 30). https://www.proquest.com/docview/2518611259/fulltextPDF/C09C72E5AA754783PQ/1?accountid=14701&parentSessionId=vA2ztSKU3sCraWt8MDFEwRvbemJVRG0t5nxTVSoDM70%3D. 10.1136/bmj.n945 33903170

[27] Canadian Nurses Association. Our leadership—Canadian Nurses Association. 2021 (cited 2021 Dec 30). https://www.cna-aiic.ca/en/membership/who-we-are/our-leadership.

[28] Bouabdillah N, Perron A, Holmes D (2021). Career advancement: the experiences of minority nurses in accessing leadership positions in a tertiary care setting. Witn Can J Crit Nurs Discourse.

[29] Jefferies K, Goldberg L, Aston M, Tomblin MG (2018). Understanding the invisibility of black nurse leaders using a black feminist poststructuralist framework. J Clin Nurs.

[30] Wildman SM, Davis AD. Language and silence: making systems of privilege visible. St CLARA LAW Rev. 35:27.

[31] Emirbayer M, Desmond M. The racial order. Chicago: University of Chicago Press; 2015 (cited 2021 Dec 31). p. 520. https://press.uchicago.edu/ucp/books/book/chicago/R/bo20069291.html.

[32] Essed P (1991). Understanding everyday racism: an interdisciplinary theory.

[33] Employment and Social Development Canada. A backgrounder on poverty in Canada—Canada.ca. (cited 2021 Nov 30). http://www.canada.ca/en/employment-social-development/programs/poverty-reduction/backgrounder.html.

[34] Statistics Canada. Changes in the socioeconomic situation of Canada’s Black population, 2001 to 2016 (cited 2021 Nov 30). http://www150.statcan.gc.ca/n1/pub/89-657-x/89-657-x2020001-eng.htm.

[35] Khan R, Apramian T, Kang JH, Gustafson J, Sibbald S (2020). Demographic and socioeconomic characteristics of Canadian medical students: a cross-sectional study. BMC Med Educ.

[36] Idriss-Wheeler D, El-Mowafi IM, Coen-Sanchez K, Yalahow A, Yaya S (2021). Looking through the lens of reproductive justice: the need for a paradigm shift in sexual and reproductive health and rights research in Canada. Reprod Health.

[37] Bailey KA (2016). Racism within the Canadian university: indigenous students’ experiences. Ethn Racial Stud.

[38] Henry F, Tator C. Racism in the Canadian University: demanding social justice, inclusion, and equity. University of Toronto Press; 2009 (cited 2021 Apr 90). 10.3138/9781442688926

[39] Glauser W. When Black medical students weren’t welcome at Queen’s. University Affairs. (cited 2021 Nov 30). https://www.universityaffairs.ca/features/feature-article/when-black-medical-students-werent-welcome-at-queens/.

[40] Jefferies K. Recognizing history of Black nurses a first step to addressing racism and discrimination in nursing. Dalhousie News. 2020 (cited 2021 Nov 30). https://www.dal.ca/news/2020/05/13/recognizing-history-of-black-nurses-a-first-step-to-addressing-r.html.

[41] DeCoteau DMA, Woods A, Lavallee DB, Cook DC. Unsafe learning environments: indigenous medical students’ experiences of racism. 8.

[42] Collie M. 24 Black medical students accepted to U of T Medicine—the most in Canadian history | Globalnews.ca. Global News. 2021 (cited 2021 Nov 30). https://globalnews.ca/news/7010646/24-black-medical-students-accepted-u-of-t-medicine/.

[43] Trinh J. Canadian doctors of colour offer a frank look at racism in medicine | CBC News. CBC. 2020 (cited 2021 Nov 9). https://www.cbc.ca/news/health/racism-canadian-medicine-doctors-1.5615554.

[44] Kassam A. Opinion | Canadian medicine has a diversity problem. The Toronto Star. 2017 (cited 2021 Nov 16). https://www.thestar.com/opinion/commentary/2017/09/03/canadian-medicine-has-a-diversity-problem.html.

[45] Asian Communities for Reproductive Justice. A New Vision. Forward Together. 2005 (cited 2021 Nov 11). https://forwardtogether.org/tools/a-new-vision/.

[46] SisterSong. Reproductive justice. SisterSOng women of color reproductive justice collective. 2021 (cited 2021 Apr 10). https://www.sistersong.net/reproductive-justice.

[47] Black Mama Matters Alliance (BMMBA). Black maternal health research re-envisioned: best practices for the conduct of research with, for, and by Black Mamas. Harv Law Policy Rev. 2020;14:394–407. https://harvardlpr.com/wp-content/uploads/sites/20/2020/11/BMMA-Research-Working-Group.pdf.

[48] Martis EE. Why black women fear for their lives in the delivery room|HuffPost null. Huffpost. 2020 (cited 2021 Nov 9). https://www.huffpost.com/archive/ca/entry/black-maternal-health-canada_ca_5ed90ae3c5b685164f2eab93.

[49] Stewart EA, Nicholson WK, Bradley L, Borah BJ (2013). The burden of uterine fibroids for African–American women: results of a National Survey. J Womens Health.

[50] McKenzie HA. Indigenous women’s reproductive (in)justice(s) and self-determination : envisioning futures through a collaborative research project. University of British Columbia; 2020 (cited 2020 Dec 22). https://open.library.ubc.ca/cIRcle/collections/ubctheses/24/items/1.0388291.

[51] FitzGerald C, Hurst S (2017). Implicit bias in healthcare professionals: a systematic review. BMC Med Ethics.

[52] Bellrichard C. Investigation finds widespread racism against Indigenous peoples in B.C. health-care system | CBC News. CBC News. 2020 (cited 2021 Dec 3). https://www.cbc.ca/news/indigenous/bc-health-care-racism-report-1.5820306.

[53] Shingler B. Investigations launched after Atikamekw woman records Quebec hospital staff uttering slurs before her death | CBC News. CBC. 2020 (cited 2021 Dec 3). https://www.cbc.ca/news/canada/montreal/quebec-atikamekw-joliette-1.5743449.

[54] Prather C, Fuller TR, Jeffries WL, Marshall KJ, Howell AV, Belyue-Umole A (2018). Racism, African American women, and their sexual and reproductive health: a review of historical and contemporary evidence and implications for health equity. Health Equity.

[55] Udonya I. Black people are not your guinea pigs. The Peak. 2020 (cited 2020 Dec 22). https://the-peak.ca/2020/07/black-people-are-not-your-guinea-pigs/.

[56] Dyck D. TRC heard concerns about coerced sterilization of Indigenous women, says Murray Sinclair | CBC News. CBC Press: The Canadian. 2018 (cited 2021 Nov 9). https://www.cbc.ca/news/indigenous/trc-concerns-coerced-sterilizations-murray-sinclair-1.4917321.

[57] Paynter MJ. Reproductive (in)justice in Canadian Federal prisons for women. Ottawa, ONT, Canada: Canadian Association of Elizabeth Fry Societies; 2021. p. 53. (2019–2020 Perproductive Justice Workshop Project). https://ac935091-bf76-4969-8249-ae3a107fca23.filesusr.com/ugd/d2d30e_13d22f66c3eb41449c2e52c519913b35.pdf.

[58] Gilliam ML, Neustadt A, Gordon R (2009). A call to incorporate a reproductive justice agenda into reproductive health clinical practice and policy. Contraception.

[59] Roberts D (1998). Killing the black body: race, reproduction, and the meaning of liberty.

[60] Rice K, Te Hiwi B, Zwarenstein M, Lavallee B, Barre DE, Harris SB (2016). Best Practices for the prevention and management of diabetes and obesity-related chronic disease among indigenous peoples in Canada: a review. Can J Diabetes.

[61] Allan B, Smylie J. First peoples, second class treatment: the role of racism in the health and well-being of Indigenous peoples in Canada. The Wellesley Institute; 2015. https://www.wellesleyinstitute.com/wp-content/uploads/2015/02/Summary-First-Peoples-Second-Class-Treatment-Final.pdf.

[62] Crowshoe L, Dannenbaum D, Green M, Henderson R, Hayward MN, Toth E (2018). Type 2 diabetes and indigenous peoples. Can J Diabetes.

